# Empirical Analysis and Modeling of Users' Topic Interests in Online Forums

**DOI:** 10.1371/journal.pone.0050912

**Published:** 2012-12-12

**Authors:** Fei Xiong, Yun Liu

**Affiliations:** 1 School of Electronic and Information Engineering, Beijing Jiaotong University, Beijing, China; 2 Key Laboratory of Communication and Information Systems, Beijing Municipal Commission of Education, Beijing Jiaotong University, Beijing, China; Cinvestav-Merida, Mexico

## Abstract

Bulletin Board Systems (BBSs) have demonstrated their usefulness in spreading information. In BBS forums, a few posts that address currently popular social topics attract a lot of attention, and different users are interested in many different discussion topics. We investigate topic cluster features and user interests of an actual BBS forum, analyzing user posting and replying behavior. According to the growing process of BBS, we suggest a network model in which each agent only replies to the posts that belong to its specific topics of interest. A post that is replied to will be immediately assigned the highest priority on the post list. Simulation results show that characteristics of our model are similar to those of the real BBS. The model with heterogeneous user interests promotes the occurrence of popular posts, and the user relationship network possesses a large clustering coefficient. Bursts and long waiting time exist in user replying behavior, leading to non-Poisson user activity pattern. In addition, the model produces an analogous evolving trend of Gini coefficients for posts' and clusters' participants as BBS forums.

## Introduction

In the last few years, complex networks and complex systems have become a research focus, attracting the attention of many researchers [1]–[3]. Researchers in this area have attempted to determine the underlying law of complex networks in real society, and to understand their essential evolutionary mechanisms. Thus, theoretical network models have been developed to simulate the actual growth process [4], [5]. These models extract the most important factor of the evolving dynamics, and therefore, they can achieve some statistical characteristics of actual networks, such as power-law degree distribution, and small average path length. On the other side, driven by data analysis, empirical studies of real networks have also been conducted, calculating the macroscopic system properties and microscopic node behavior [6]–[9]. Many networks have been investigated, such as social networks [10], airport networks [11], protein elastically bonded networks [12], and scientific collaboration networks [13]. Some phenomena in these networks have been identified and explained. Barabasi and Albert proposed a network model, the degree distribution of which follows a power law [14]. In their model, new nodes are added to an initially small-scale network, and for each new node, the probability of connecting to old nodes is proportional to the degrees of the old nodes. Therefore, the longer a node has been in the network, the more connections it is likely to have. The clustering coefficient of the network decays rapidly as the size of the system increases. Watts and Strogatz constructed a small-world network by breaking and reconnected the edges of a regular network at random [15]. This network has both a small average shortest path length and a large clustering coefficient even with large system size, but the degree does not have a power-law distribution.

With the development of computer technology, analysis and computing of large-scale real data have been taken into consideration. In Ref. [16], the degree distribution of the World Wide Web decays strictly as a power law, and there is no correlation between the node age and node degree. Similar topological features and evolving pattern of node degrees were found in the text message network of mobile phones, but the intrinsic growth process was quite different [17]. In the text message network, the dynamics is dominated mainly by the local-preference connecting protocol. On the contrary, the Chinese air route network has an exponential degree distribution and a low clustering coefficient. In addition, flight flow on this network is considerably heterogeneous [11]. Furthermore, the assortativity coefficients were explored both in the blogging network and social networking site (SNS), proving that the bidirectional SNS is an assortative network while the directional blogging network is disassortatively mixed [18]. These studies on real networks can help understand the dynamics on these systems, including rumor spreading, package communication, and opinion evolution.

Internet has become the main place for people to get information because it provides a free and convenient way to browse and search for information. In Web 2.0 networks that are self-organized, website contents are created by users, and user interactions promote the growth of networks. As a typical representative of Web 2.0, the BBS forum becomes an open place where information can be diffused. In the BBS forum, each registered user can publish posts to express its ideas and emotions. After a user creates a post, other registered users can publish replies to the post. Meanwhile, there are often a great many latent users that read posts but never publish replies. Since it is hard to collect the information of latent users, only the users that publish posts or replies are considered as participants in the forum. As far as we know, the BBS forum is a scale-free and small-world network, possessing both a power-law degree distribution and a large clustering coefficient [19]–[21]. In addition, the dissipative behavior of users in BBS was also been investigated [22]. However, there are still some questions about BBS that need to be answered. A few posts have many replies and participants, but most posts attract less interests and fewer replies. This raises the question concerning whether the popularity of a post is correlated with its content. Indeed, we find posts about some social events, such as food safety, readily attract a lot of attention. We also observe that in BBS forums people concentrate only on the posts that interest them, and users' topics of interest are quite heterogeneous. In this paper, we present a growth model driven by reading and replying to posts according to user interests. Each user only replies to the posts that fall within its topics of interest. We show the topic cluster and topological characteristics of an actual BBS forum, and compare this BBS with our model.

The rest of the paper is structured as follows. Section 2 analyzes the topic characteristics of a real BBS. In Section 3 we put forward our model in terms of the growing mechanism of BBS. Simulation results and discussions of the model are given in Section 4. We close the paper in Section 5 with concluding remarks.

## Analysis of Actual Data

The Tianya BBS (www.tianya.cn) is one of the most famous BBS forums in China. Since March 1999, the Tianya BBS has attracted more than 60 million registered users and a huge number of anonymous users. As a result of its numerous participants, Tianya BBS is often the public place of fierce debates on some social events, and sometimes the source of online emergency. Some posts that are discussed originally by several users in local scope, have caused public response, and their influence has exceeded the local circumstance. Therefore, research on Tianya BBS can shed valuable light to understand network evolution and information diffusion on social media in the age of Web 2.0, and it also can support the early warning of online emergency. We collected data from the Tianya BBS's economic board which is extremely related to people's daily lives. Posts, replies and users' information were crawled by our directed robot. In all, we acquired 10971 posts and 315464 replies, including 90276 corresponding users.

As far as we know, the distribution of replies for each post and for each user decays as a power law [19], meaning most of users only participate in a few posts. Meanwhile, even posts that are created at approximately the same time, often address different topics. Since our data were downloaded from the economic board, these topics describe some economic or social events occurring in real society, such as the economic crisis, economic crime, and stock investment. For instance, some posts explain the reasons for the 2008 financial crisis; some posts characterize the influence of that crisis including unemployment of workers and bankrupting of companies; and some posts discuss possible solutions and policies for dealing with the crisis. Therefore, these posts describe the same event and belong to the same topic. It is obvious that different topics attract different people. Topics closely related to the issues that people face in their daily lives are very popular, such as the economic crisis, and taxes and tariffs, while other topics, such as economic data analysis, are not among people's major concerns.

Users may participate in posts about different topics, and these topics reflect their interests and preferences. We cannot collect the readers of posts, so topics of user's implicit interest are confirmed based on whether the user replies to a post about specific topics. Since users have various levels of interests, we try to detect user's interest by clustering topics among all of the posts. Texts in some languages do not use separative signs to split terms, so firstly we should split the continuous text of each post's content into separate terms [23], and transfer the post to a term vector. In the term vector, each term in a post is represented as a weight value that is measured by the traditional 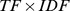 definition, according to the number of times the term occurs. The similarity between two posts is defined as the cosine distance between the corresponding term vectors. Then, the K-Means algorithm [24] is used for clustering topics, and posts that are very similar to each other are assigned to the same cluster. We set the number of clusters 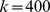, and this quantity 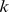 is approached by text databases [25]. We repeat the clustering 10 times, each with a new set of initial random centroids. We also use Carrot2 tools with Lingo algorithm [26] to test k. For 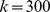, some posts are still unlabelled in the group of “Other topics” after the clustering, while for 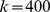, few posts are left without a cluster label.

**Figure 1:**
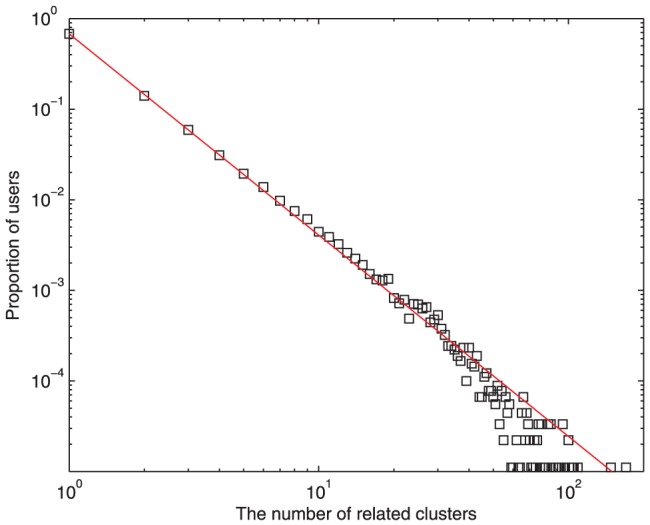
Distribution of the number of users' topic interests in Tianya BBS. The double logarithmic curve shows a power-law property, with the power exponent of 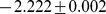. Most users concentrate on less than 10 topics, and none of users have more than 200 topic interests.

**Figure 2:**
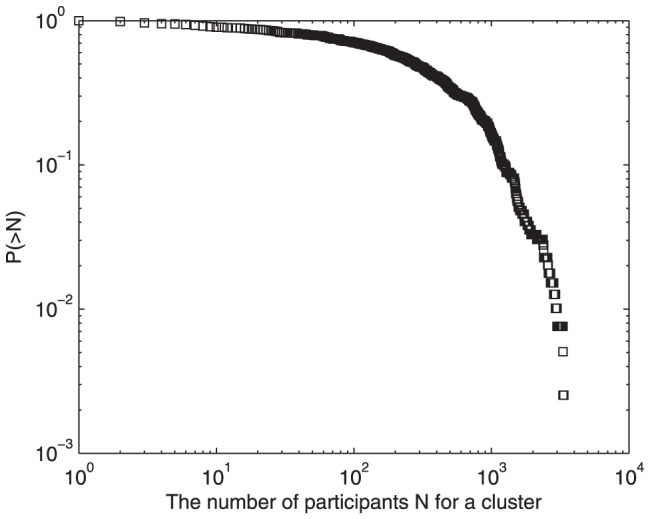
Cumulative distribution of the number of participants for each topic cluster in Tianya BBS. About 90 percent of clusters attract more than 10 participants, indicating some of the least popular topics can still have some followers. Meanwhile, when 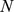 is large, the probability curve decreases significantly with the increase of 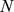, and only a tiny fraction of topics attract several thousand users. The intermediate quantity 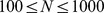 occupies at least 80 percent of topic clusters, implying 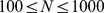 is the common pattern of participants in a topic cluster.

After clustering the topics, each post is assigned a cluster label. We calculate the number of topics in which a user participates. Figure 1 shows the distribution of this number for each user. The double logarithmic curve shows a power-law property, with the power exponent of 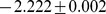. Most users concentrate on less than 10 topics, and it is rare for users to have a wide range of topic interests. Moreover, none of users have more than 200 topic interests. In terms of their interests, users may reply to several posts about a topic, or they can join in discussions about different topics. The heterogeneity of users' interests implies different user participation activity, probably leading to an asymmetric user distribution for each cluster. As shown in Fig. 2, the number of users 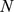 participating in each cluster is counted, and the users' cumulative distribution is determined. Clearly, the distribution curve has an exponential decaying approximately, unlike the power-law distribution of users' topics of interest. About 90 percent of clusters attract more than 10 participants, indicating some of the least popular topics can still have some followers. Meanwhile, when 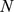 is large, the probability curve decreases significantly with the increase of 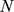, and only a tiny fraction of topics attract several thousand users. The intermediate quantity 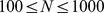 occupies at least 80 percent of topic clusters, implying 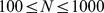 is the common pattern of participants in a topic cluster.

**Figure 3:**
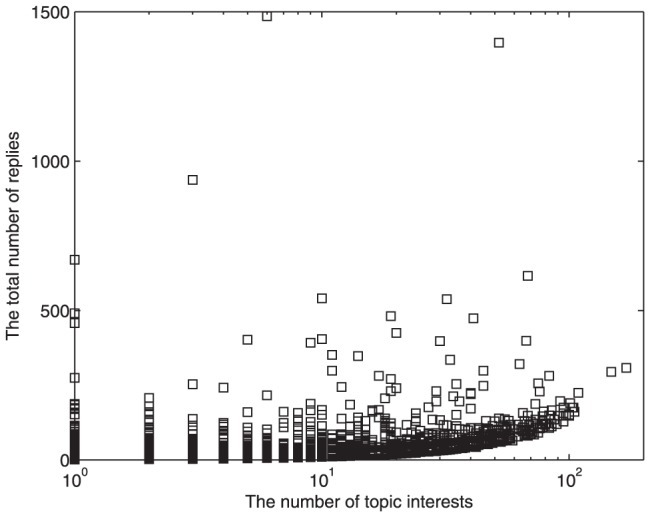
Total number of replies for each user as a function of the number of user's topic interest. Some users that are interested in many topics usually create several replies for each topic and never join in further discussions. On the contrary, some users concentrating on only a few topics might debate with others and publish many replies to support their own opinions. The most active user that publishes about 1500 replies is only interested in 6 topics.

Intuitively, users that have a wide range of topic interests may be quite active and are likely to publish many replies. However, as illustrated in Fig. 3, although the lower bound of number of replies increases slowly with the number of user's topic interest, topic interests are not the crucial factor of users' activity. Some users that are interested in many topics only create several replies for each topic and never join in further discussions. On the contrary, some users concentrating on only a few topics might debate with others, and publish many replies to support their own opinions. It is observed that several enthusiasts even reply more than 500 times to the posts about an identical topic. The most active user that publishes about 1500 replies is only interested in 6 topics.

**Figure 4:**
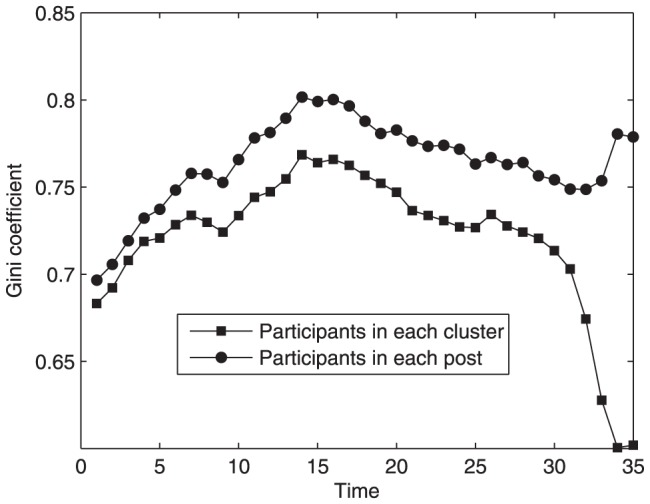
Gini coefficient for the distribution of participants in each topic cluster and in each post as a function of time in Tianya BBS. The two Gini coefficients increase quickly in the early stage. However, the Gini coefficient for posts' participants eventually tends to become relatively stable, while the Gini coefficient for clusters' participants may decrease markedly.

The Gini coefficient is a measure in economics and ecology to characterize the inequalities in the distribution of resources [27]. We use this coefficient to determine the nonuniform distribution of participants among each topic cluster. We acquire the Lorenz curve of the distribution in the following way [28]. We calculate the number of participants 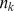 in cluster 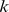 until a given time. Let 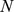 denote the total number of participants until the given time and 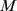 denote the number of clusters; then, we sort all of the clusters in increasing order of the proportion 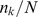. We label these ordered clusters as 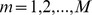, and then, the Lorenz curve is attained by 
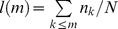
. Therefore the Gini coefficient is defined as the area ratio between the Lorenz curve for distribution of clusters' participants and the Lorenz curve for uniform distribution 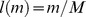. We choose 35 continuous months from March 2006 to January 2009 and calculate the Gini coefficient for the distribution of clusters' participants month by month. Analogously, the Gini coefficient for posts' participants is also depicted in Fig. 4. The two Gini coefficients increase quickly in the early stage, indicating that the difference of popularity between these clusters or posts is being enlarged rapidly. Since popular clusters or posts have a large quantity of potential followers, more and more users probably become involved in these clusters or posts. Therefore, the growth of a few popular clusters or posts makes the Gini coefficient increase. After that stage, the Gini coefficient for posts' participants eventually tends to become relatively stable with some small fluctuations. In this stage, the growth of existing popular posts improves the Gini coefficient, while the gradual emergence of new popular posts and the slow growth of older, less popular posts decrease the Gini coefficient. The effects of these opposite factors are comparable. However, the Gini coefficient for clusters' participants may decrease markedly, because the topic clusters that are not very attractive also can generate popular posts discussed in the global context. In addition, new users that prefer less popular clusters can contribute unattractive posts into these clusters, resulting in a phenomenon that the Gini coefficient for posts' participants keeps stable while the coefficient for clusters' participants may decrease. Furthermore, we find that, in addition to the cluster feature, there are also some other related features of post's popularity, such as user activity and the number of other potentially popular posts at the current time. We will consider these features in post evolving mechanism.

## The Model

In Ref. [29], the authors assumed that users will skip the posts they have already read and posts are ranked by the creating time, so a user cannot reply to a post more than once. Actually, in the BBS forum, the rank of posts is changed both for creating and replying to posts. The system places a new post that has just been created at the top of all posts in the board of the forum, so this post is more likely to be noticed by other users. Meanwhile, after a user replies to an existing post, the post is also moved to the top of posts. Therefore, these posts that are replied to frequently are usually located in the front and well-marked place on the webpage, so they can attract more users. This is the so-called “rich-get-richer” concept. However, users read posts from the top to the bottom of the webpage, and they only join in the posts that attract their interests. Posts that are not interesting to the users will be skipped, and the interests of current, active users have a significant influence on the growth of posts. If there are not enough followers at a given time, some posts that belong to popular topic clusters may be neglected. In the following, we introduce our model based on user interests.

In the BBS forum, there is no predefined network topology that describes the relationships between users. In fact, users build a connection on the condition that they reply to the same post. Therefore, user relationships are constructed when sharing their interests during the interactions. This means the network topology for gaining information has higher connectivity than that for opinion updating [30]. Assume there are a total of 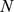 potential agents that are not initially connected, and each of them has its own topic interest. The distribution of users' topic interests decays as a power law consistent with Tianya BBS (Fig. 1.), and the power exponent is 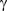. In an action event, an agent is selected at random. According to its topic interest, it may publish a new post about one of its topics of interest with a probability 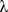. Meanwhile, the agent will read the existing posts from top to bottom and reply to the posts that satisfy its topic interest. A post that is created or replied to at the present time is put at the top of the post set. Since the agent is not energetic enough to concentrate on all posts, its inclination for continuing to read posts decays with time. Therefore, after the agent reads a post, it may drop out of this action event with a probability 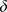. Then the action event is repeated for a certain number of times. In this model, a post may be replied to by the same user more than once if the post is popular enough.

A previous study [31] determined that user activity is promoted by controversial emotions. Two users reply to a post frequently when they hold opposite opinions towards this post, and they may debate fiercely. These posts in which more divergence among users' opinions exists are likely to have more replies. Indeed, in many popular posts, two or more opposite opinions struggle against each other. However, we also find some popular posts in Tianya BBS that approximately form a consensus opinion. For instance, posts about economic crime often have a large number of replies, and most people show indignant and disgusting emotions towards the perpetrators of the event. This means that this kind of event usually gains public notice. Therefore, users' interests play a significant role in users' participation decisions and the popularity of posts.

## Simulation Results and Discussion

We carry out numerical simulations of our model with varied parameters to investigate the elaborate process of post's growth and regression and to analyze the heterogeneity of topic occurrence and user participation. In an action event, an agent is picked randomly to participate in the process of reading and publishing posts according to the rules mentioned above. For an 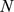-size system, the time step is increased by 1 after 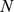 such action events. In the beginning agents' topic interests are initialized, and the number of agent's topics follows a power-law distribution. We set the power exponent of topic interest distribution at 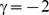, analogously to that of Tianya BBS. We also check other power exponents around the actual value, and gain similar results. Meanwhile, for an agent, topic interests are selected following a power law with a small exponent −0.3 to ensure a few public topics. The parameter 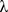 denotes the probability that a user publishes a new post each time. We choose a tiny 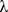 to make the proportion between posts and replies comparable with that of actual BBS. The proportion between posts and replies in Tianya BSS is 0.0348, while in our model for 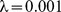 and 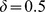, the proportion is 0.025.

### Post and Reply Behavior

**Figure 5:**
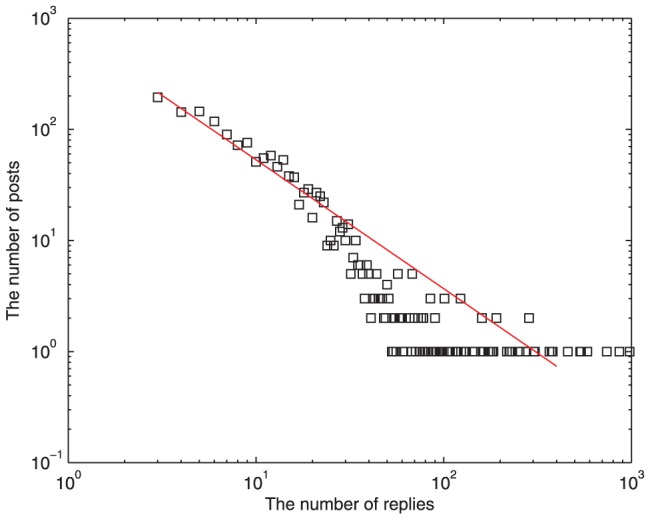
Number of posts varying their number of replies after 200 time steps, 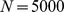, 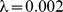, and 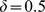. This distribution decays as a power law along with a long tail. The power exponent is 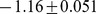.

**Figure 6:**
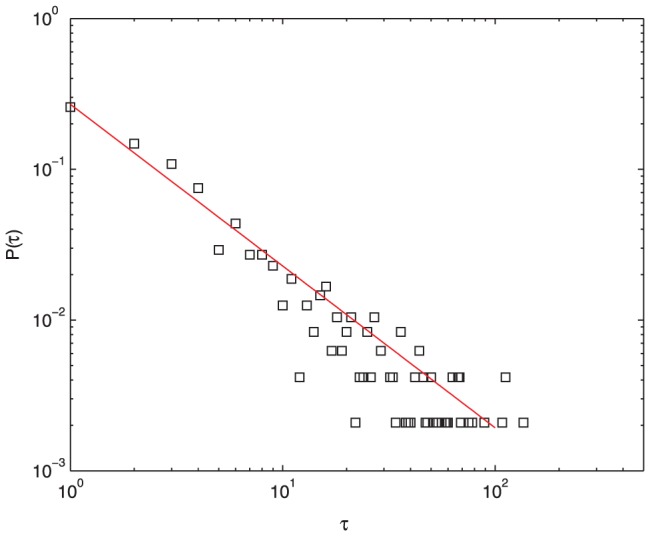
Distribution of waiting time between consecutive threads published by a typically selected user after 5000 time steps, 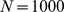, 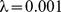, 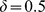. The distribution of the time differences between consecutive threads published by a typically selected user is approximated by 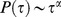 where 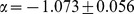, indicating that a user activity pattern has non-Poisson statistics.

Figure 5 shows the heterogeneity of the number of replies for different posts. In accordance with previous studies [19], this distribution decays as a power law along with a long tail. From this chart, it is concluded that the nonuniform levels of posts' attraction are caused by the variety of users' topic interests. From Fig. 6, the distribution of the time differences between consecutive threads published by a typically selected user is approximated by 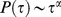 where 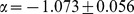, indicating that a user activity pattern has non-Poisson statistics. A user may publish threads frequently in a short succession, followed by a long period of waiting time in which no posts and replies are created by the user. This long tailed interevent time distribution agrees with the studies reported in [32], [33], which introduced a priority queue process for modeling individual decision making. In our situation, each user shares the same priority list of available posts. Users will consider the highly prior posts on the list, and only reply to these posts belonging to their topics of interest. The difference between our model and Barabasi's work is that in our model, the priority list may be changed after a user replies to a post. Therefore, this kind of priority selection mechanism enforces bursts in posting and replying pattern, and leads to non-Poisson dynamics. Moreover, the absolute value of the exponent 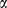 increases with the number of user's topics of interest, but it decreases with the increase of 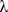 or 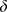.

### Topic Characteristic

**Figure 7:**
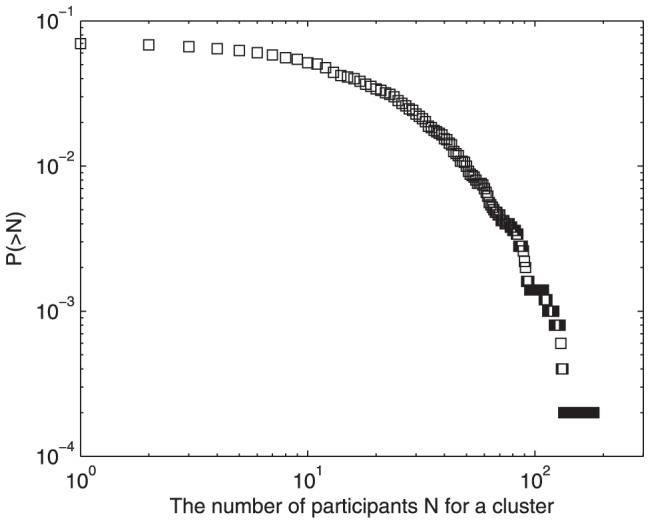
Cumulative distribution of the number of participants for each cluster after 200 time steps, 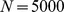, 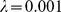, and 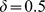. This curve decays more and more rapidly in the double logarithmic coordinates. This distribution coincides with the Tianya BBS in Fig. 2.

**Figure 8:**
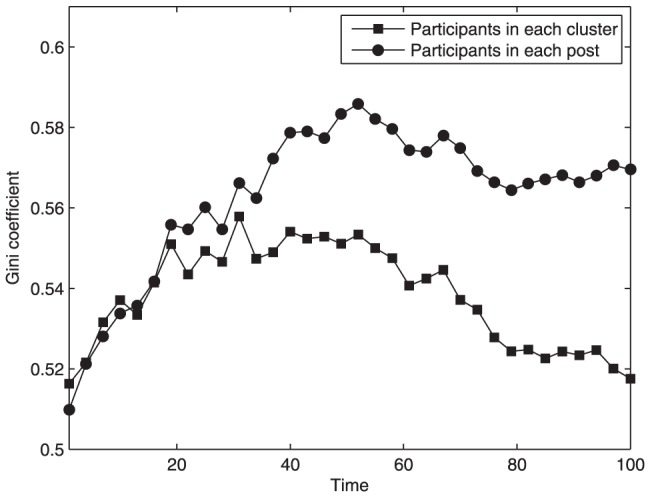
Gini coefficient for the distribution of participants in each cluster and in each post of our model as a function of time, 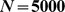, 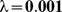, 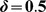. The Gini coefficient for posts' participants remains relatively stable after a rapid increase, but the coefficient for clusters' participants obviously decreases with the time elapsed.

Figure 7 illustrates the cumulative distribution of participants in each topic cluster. It is obvious that this curve decays more and more rapidly in the double logarithmic coordinates. This distribution coincides with the Tianya BBS. Figure 8 shows the evolving trend of Gini coefficients for the number of participants in each cluster and in each post during a period of continuous time steps. The Gini coefficient for posts' participants remains relatively stable after a rapid increase, but the coefficient for clusters' participants obviously decreases with the time elapsed. The behaviors of Gini coefficients do not depend on the initial condition. Although the Gini coefficients change with the parameters and distribution of users' topic interests, the time evolutionary trend of Gini coefficients is similar. In the rich-get-richer system such as Barabasi-Albert network, the Gini coefficient increases with time at first, and then it will reach a plateau. Therefore, the preferential attachment mechanism makes the Gini coefficient increase. On the other side, the decrease of Gini coefficient for clusters' participants is caused by the occurrence of popular posts in less popular clusters and the increase of total number of posts in those clusters.

### User Relationship

**Figure 9:**
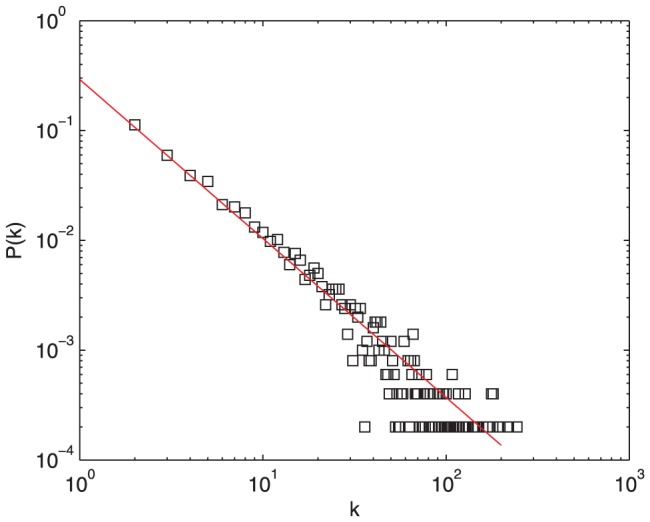
Degree distribution of our user network, 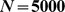, 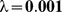, 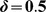. User nodes have a power-law degree distribution with the power exponent 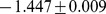.

In BBS forums, relationships exist between post creators and repliers. When users participate in a post, they can communicate with each other, expressing their own ideas to support or argue with other users. Connections are conceived to be constructed after a user replies to another user's post. Therefore, users denote the nodes in the network and links between a post and a reply can be treated as edges. Then we analyze the topological features of the user network created by our model. In Fig. 9, user nodes have a power-law degree distribution, which is consistent with that of actual BBS forums [20]. The power exponent of degree distribution is 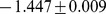. The clustering coefficient of user topology is 0.2017 for 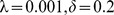, and 0.1570 for 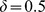. It was found that the clustering coefficient for Tianya BBS is 0.111, so our user network has high clustering just as the actual system. Therefore our user network has scale-free properties, and it also displays as a small-world network.

## Conclusions

In BBS forums, posts that address different topics have unequal attraction for users. In this paper, we analyzed topic cluster features of the Tianya BBS. We used machine learning algorithm to detect user interests, calculated the distribution of clusters' participants, and determined the evolutionary trend of Gini coefficient. We found that the number of user topic interests has a nonuniform distribution. We proposed a growth model that simulates user posting and replying behavior in BBS forums. Users are initialized with different topics of interest, and users only reply to the posts in which they are interested. We also carried out numerical simulations and compared the results with those of the Tianya BBS.

Simulation results show that due to heterogeneous user interests, the number of replies for each post decays as a power law, implying the occurrence of a few very attractive posts. The distribution of time intervals between consecutive threads for a user does not follow a Poisson process, and long periods of waiting time exist between user replying behavior. The Gini coefficient for clusters' participants may decrease significantly with time. Moreover, user relationship topology is also analogous to the real BBS. In future work, we will combine our model with data mining method, and develop a predictive model to detect popular posts in their early stages.

## Methods

### Ethics Statement

This study does not need the approval of ethics committee, since this work does not include any sensitive or ethical content. This work is related to BBS forum that is a kind of online social networks, and this work uses statistical physics and computer methods to determine the macroscopic properties.

This work does not need the consent of participants for their information in Tianya BBS, because the BBS forums including Tianya are an open place, where people can freely read and download author names and the contents of posts and replies. On the Tianya website, only registered users can publish posts and replies, and they must login the website using their usernames and passwords; however, everyone can freely read the information on Tianya. In Tianya BBS, users' personal profiles including their usernames and passwords are private information and cannot be seen by any other user, but posts, replies and names of authors are free and open information. There is a button “Browse and enter” on the mainpage of Tianya website. By clicking this button, everyone can anonymously enter the forum without logining, and can read the posts and replies freely.

In our work, we only need post and reply information, and names of authors, and all of these are not related to any personal profile. We downloaded these data by our directed robot freely based on HTTP protocol just as people browse the BBS freely. Therefore, there is no need to gain the permission from Tianya to use the data, since the BBS forum provides the data freely to the public. Everybody can read and download these data freely.

All the data were anonymized for analysis. The BBS forum does not have the real-name authentication. Everyone can register its account using an arbitrary username without the need to use its real name in society. Therefore, the names of authors we downloaded from Tianya are not users' real names and are not private information. Meanwhile we only used the names of authors as a sign to distinguish different users, so we anonymized all the data.
